# Detección y expresión de SapS, una fosfatasa ácida no específica de clase C con actividad de fosfatasa O-fosfotirosina, en aislamientos de *Staphylococcus aureus* de pacientes con osteomielitis crónica

**DOI:** 10.7705/biomedica.6604

**Published:** 2023-06-30

**Authors:** Carlos Martínez-Canseco, Rebecca E. Franco-Bourland, Norma González-Huerta, Marco Antonio Paredes-Espinosa, Silvia Giono-Cerezo, Laura Sánchez-Chapul, Rogelio Paniagua-Pérez, René Valdez-Mijares, Cecilia Hernández-Flores

**Affiliations:** 1 Servicio de Bioquímica, Instituto Nacional de Rehabilitación “Luis Guillermo Ibarra Ibarra”, Ciudad de México, México Instituto Nacional de Rehabilitación Servicio de Bioquímica Instituto Nacional de Rehabilitación “Luis Guillermo Ibarra Ibarra” Ciudad de México Mexico; 2 Servicio de Medicina Genómica, Instituto Nacional de Rehabilitación “Luis Guillermo Ibarra Ibarra”, Ciudad de México, México Instituto Nacional de Rehabilitación Servicio de Medicina Genómica Instituto Nacional de Rehabilitación “Luis Guillermo Ibarra Ibarra” Ciudad de México Mexico; 3 Servicio de Bioterio y Cirugía Experimental, Instituto Nacional de Rehabilitación “Luis Guillermo Ibarra Ibarra”, Ciudad de México, México Instituto Nacional de Rehabilitación Servicio de Bioterio y Cirugía Experimental Instituto Nacional de Rehabilitación “Luis Guillermo Ibarra Ibarra” Ciudad de México Mexico; 4 Laboratorio de Bacteriología Médica, Escuela Nacional de Ciencias Biológicas, Instituto Politécnico Nacional, Ciudad de México, México Laboratorio de Bacteriología Médica Escuela Nacional de Ciencias Biológicas, Instituto Politécnico Nacional Ciudad de México México; 5 Laboratorio de Enfermedades Neuromusculares, Instituto Nacional de Rehabilitación “Luis Guillermo Ibarra Ibarra”, Ciudad de México, México Instituto Nacional de Rehabilitación Laboratorio de Enfermedades Neuromusculares Instituto Nacional de Rehabilitación “Luis Guillermo Ibarra Ibarra” Ciudad de México Mexico

**Keywords:** Staphylococcus aureus, virulence factors, osteomyelitis, Staphylococcus aureus, factores de virulencia, osteomielitis

## Abstract

**Introducción.:**

Se desconoce la identidad de los factores de virulencia de *Staphylococcus aureus* implicados en la osteomielitis crónica. Sin embargo, SapS, una fosfatasa ácida no específica de clase C, es un factor de virulencia reconocido y ya fue identificada en la cepa 154 de *S. aureus,* pero en extractos proteicos de vegetales podridos.

**Objetivo.:**

Detectar el gen *SapS* y caracterizar la actividad de la fosfatasa SapS en cepas de *S. aureus* aisladas de pacientes con osteomielitis crónica y en las reportadas en una base de datos de análisis *in silico* de genomas bacterianos completos*.*

**Materiales y métodos.:**

Se aisló y secuenció el gen *SapS* en los 12 aislamientos clínicos de *S. aureus* y en dos cepas de referencia; estas secuencias se analizaron junto con las secuencias de las cepas reportadas en la base de datos de genomas bacterianos: 49 cepas de *S. aureus* y 11 cepas de estafilococos negativos para coagulasa. Se evalúo la actividad de la fosfatasa SapS, presente en los extractos de los sobrenadantes de los cultivos de las cepas clínicas, mediante la hidrólisis de fosfato p-nitrofenil, O-fosfo-L- tirosina, O-fosfo-L serina y O-fosfo-L treonina junto con varios inhibidores de fosfatasas.

**Resultados.:**

Se detectó el gen *SapS* en el genoma de las cepas clínicas y en las 49 cepas de *S. aureus* analizadas *in silico*, pero no en las 11 cepas de estafilococos negativos para coagulasa. La secuenciación de *SapS* reveló un péptido señal presente en el extremo N-terminal de proteínas extracelulares y los dominios bipartitos de aspartato (DDDD) en su sitio catalítico. SapS hidroliza selectivamente el fosfato p-nitrofenil y la O-fosfo-L-tirosina, pero es sensible a vanadato y molibdato.

**Conclusión.:**

Se encontró *SapS* en el genoma de *S. aureus* de las cepas clínicas y de las cepas de simulación computacional. La SapS con actividad específica para la hidrólisis de la O-fosfo-L-tirosina comparte similitudes bioquímicas con las fosfatasas-tirosina bacterianas, por lo que puede formar parte de la red de factores de virulencia de la osteomielitis crónica.

## Abstract (en)

**Introduction.:**

The identity of *Staphylococcus aureus* virulence factors involved in chronic osteomyelitis remains unresolved. SapS is a class C non-specific acid phosphatase and a well-known virulence factor that has been identified in *S. aureus* strain 154 but in protein extracts from rotting vegetables.

**Objective.:**

To identify the *SapS* gene and characterize the activity of SapS from *S. aureus* strains: 12 isolates from bone infected samples of patients treated for chronic osteomyelitis and 49 from a database with *in silico* analysis of complete bacterial genomes.

**Materials and methods.:**

The *SapS* gene was isolated and sequenced from 12 *S. aureus* clinical isolates and two reference strains; 49 *S. aureus* strains and 11 coagulase-negative *staphylococci* were tested using *in silico* PCR. Culture media semi-purified protein extracts from the clinical strains were assayed for phosphatase activity with *p*-nitro-phenyl- phosphate, O-phospho-L-tyrosine, O-phospho-L-serine, and OphosphoL-threonine in conjunction with various phosphatase inhibitors.

**Results.:**

*SapS* was detected in the clinical and *in-silico S. aureus* strains, but not in the *in silico* coagulase-negative *staphylococci* strains. Sec-type I lipoprotein-type N-terminal signal peptide sequences; secreted proteins, and aspartate bipartite catalytic domains coding sequences were found in the *SapS* nucleotide and amino acid sequence analysis. SapS dephosphorylated with *p*-nitro-phenyl-phosphate and ophosphoLtyrosine were selectively resistant to tartrate and fluoride, but sensitive to vanadate and molybdate.

**Conclusion.:**

*SapS* gene was found in the genome of the clinical isolates and the *in silico S. aureus* strains. SapS shares biochemical similarities with known virulent bacterial, such as protein tyrosine phosphatases, suggesting it may be a virulence factor in chronic osteomyelitis.

*Staphylococcus aureus* is an opportunistic pathogen for humans and the main cause of osteomyelitis, which can evolve from an acute to a chronic stage characterized by bone loss and destruction. The temporary or permanent disability that ensues, in addition to substantial increases in hospitalization times and healthcare costs, are heavy social and institutional burdens ^1^^,^^2^.

*Staphylococcus aureus* pathogenicity is due to the plasticity of its genome and the expression of an arsenal of virulence factors ^3^^,^^4^. The identity of active *S. aureus* virulence factors in chronic osteomyelitis remains unresolved. Although the focus is currently on discovering new factors, there is also interest in exploring the virulence of well-known *S. aureus* factors such as protein A, Panton-Valentine leukocidin, and coagulase, that recently have shown to play a role in bone loss and destruction in osteomyelitis ^5^.

Various genera and species of prokaryotes express non-specific acid phosphohydrolases (NSAP), class A, B, and C, that can be attached to cell membranes or are soluble extracellular enzymes capable of “non-specifically” dephosphorylating a broad range of unrelated organic substrates. Initially, this was understood as a mechanism to acquire inorganic phosphate, an essential substrate for bacterial metabolism. However, further evidence proved that these phosphatases play a role in bacterial pathogenicity ^6^^,^^7^.

Du Plessis *et al*. ^8^ characterized a soluble acid phosphatase (SapS) from the culture supernatant of *S. aureus* (strain 154) isolated from rotting vegetables. The phosphatase is magnesium chloride dependent and EDTA and sodium molybdate can affect its function. The corresponding *SapS* gene encodes a protein with an estimated molecular mass of 30 kDa and 296 amino acids with a 31-residue signal peptide; four conserved sequence motifs were identified with structural homology to the bacterial class C family NSAPs.

Our aim was to investigate the presence of the *SapS* gene in 12 strains of *S. aureus* isolated from infected bone samples of patients treated for chronic osteomyelitis and in 49 *S. aureus* strains from a database containing *in silico* complete bacterial genomes, determine SapS presence and activity in partially purified protein extracts from supernatants of the clinical strains culture media, and compare them with those previously described ^8^.

The SapS gene was present in the 12 *S. aureus* clinical strains, *in silico* the 49 strains, but not in the 11 coagulase-negative strains. SapS presents N-terminal domain and belongs to the *S. aureus* system Sec-type I system of extracellular proteins and their bipartite aspartate (DDDD) catalytic domain.

Partially purified SapS of clinical strains was found to dephosphorylate O-phospho-tyrosine but not O-phospho-serine or O-phospho-threonine and it was resistant to sodium tartrate, but sensitive to tyrosine phosphatase inhibitors.

Our results show that the *SapS* gene was present in the clinical strains genome and that extracellular acid phosphatase preferentially dephosphorylate O-phospho-tyrosine suggesting that during chronic osteomyelitis this enzyme may interact with bone tissue cells favoring pathological bone resorption. Detailed studies should be conducted to elucidate its possible involvement in bone pathogenicity.

## Materials and methods

### Isolation and identification of Staphylococcus aureus clinical strains

The 12 *S. aureus* strains under study were isolated between 1997 and 1998 from infected bone chips (6-10 mm^2^, approximately) of adult patients during surgical debridement treatment of tibiae and femurs in the Bone Infection Service at the, then National Institute of Orthopedics, now the National Institute of Rehabilitation in Mexico City.

Bone specimens were washed twice and suspended in 1.0 ml of phosphate- buffered saline, grinded for one minute in a tissue grinder, and centrifuged at 4000*g*. Supernatants were streak-seeded in blood agar media, mannitol salt phenol red agar (Merck), and Baird-Parker agar (Merck), and incubated aerobically at 37 °C for 24 h. Characteristic bacterial isolates were identified using the standard biochemical tests and antibiotic susceptibility for *S. aureus* (Uniscept 20GP System). Gram staining, coagulase (Bactident Coagulase, Merck), and catalase (Catalase Assay Kit, Merck) assays were performed. Clinical strains were methicillinsensitive and were grouped according to infection description: chronic osteomyelitis (17 CPJ, 69 GGT, and 92 HM); infected pseudarthrosis (54 SL, 68 FFC, 88 VTM, 89 RTC, and 93 EMC); and chronic bacterial osteitis (76 IQM, 101 AOC, 105 IMO, and 107 FMR).

Clinical and reference strains (*S. aureus* ATCC-6538 and *S. epidermidis* ATCC12228) were preserved in casein peptone-soymeal peptone broth (CASO, Merck) in a solution with 10 % of glycerol at -80 °C.

### Bacterial DNA extraction

Genomic DNA was extracted as described by Novick (9) with modifications. Bacterial suspensions grown in brain-heart infusion medium (value of absorbance at 620 nm = 0.8 ) were centrifuged for five minutes at 10,000*g*; bacterial pellets were resuspended in 20 pl of a mixture of lysostaphin-lysozyme/ Tris-EDTA buffer (TE) at a final concentration of 5.0 pg/pl each (Sigma Chem). After 30 minutes at 37 °C, DNA was extracted using the Genomic DNA Purification Kit (Promega) and resuspended in 50 pl of TE buffer; purity was tested by agarose gel electrophoresis and concentration by ultraviolet (UV) spectroscopy. Extracted DNA was stored in 20 pl aliquots at -20 °C.

### SapS gene molecular detection

*SapS* gene (accession number AY061973) was detected in genomic DNA extracts by end-point polymerase chain reaction (PCR) with the oligonucleotide primers *SapS-*forward (FW) 5’ - GGCATGAATAAAATTTCAAAG 3’ and *SapS*-reverse (RV) 5’ GGCTGCAGTTATTTAACTTCGCCTGT - 3’, as previously reported (5). The expected fragment size was 891 base pairs (bp) in the location 1-891.

We also amplified the nucleotide sequence encoding the mature extracellular peptide with acid phosphatase activity using oligonucleotide primers designed by us with the Primer3 software, named *SapS2*- FW 5’ CCAAAAGTTCTGCTGAAGTTC - 3’ and *SapS2*-RV 5’ TTATTTAACTTCGCCTGTTTT 3>. The expected fragment size was 800 bp in the location 92-892.

The reaction mixture (20 pl) consisted of 30 ng of target DNA, 200 pM of deoxynucleotide triphosphates (dNTP) (100 mM dNTP set, Invitrogen), 0.5 pM of each oligonucleotide primer pair, 2.0 mM of MgCl_2_, and 1.0 unit of thermostable DNA polymerase (Platinum Taq DNA Polymerase, Invitrogen). Amplifications were performed in a thermal cycler (Eppendorf Mastercycler gradient) programmed as follows: an initial 3-minute cycle at 94 °C, 35 cycles of 1-minute steps at 94 °C, 52 °C and 72 °C, and a final 5-minute cycle at 72 °C. After the agarose gel electrophoresis, PCR products were analyzed under UV transilluminator device.

### SapS gene detection in Staphylococcus aureus and coagulase-negative staphylococci strains from the database of complete bacterial genomes

An *in-silico* PCR was performed with the DNA sequences of 49 *S. aureus* and 11 coagulasenegative *Staphylococcus* strains found in the database, with *in silico* analysis of complete bacterial genomes, reported by Bikandi and peers (10). We used the same primer pairs described above. However, given the requirements to fill out the FASTA format for the *SapS* gene, restriction sites for both forward (FW: 5’ GGC 3’) and reverse (RV: 5’ GGCTGCA 3’) primer sequences were deleted. Thus, the trimmed primers used for *in silico SapS* amplification were FW: 5’ - ATGAATAAAATTTCAAAG - 3’ and RV: 5’ - GTTATTTAACTTCGCCTGT - 3’.

Experiments were designed to allow two nucleotide mismatches at the 5’ end, no mismatches at the 3’ end, and a maximum number of 3,000 nucleotides for the amplified fragments. Results were obtained 60 seconds after the starting program. A screen capture was made showing fragment amplifications with their molecular sizes.

### Sequencing of PCR products and molecular analysis of SapS gene

Sequencing was performed with an Abi Prism 310 sequencer (Genetic Analyzer, Applied Biosystems) using a commercial reagent kit (BigDye Terminator v3.1 Cycle Sequencing Kit, Applied Biosystems). We used 120 ng of each PCR product as DNA template, and 0.5 pM of the SapS forward primer. Electropherograms and nucleotide sequences were stored and analyzed using the Chromas V2 program.

A comparative multiple nucleotide sequence paired global alignment was performed among the nucleotide sequences, and also with the *SapS* coding sequence (GenBank® AY061973) (5), using the Clustal Omega program ^11^. The nucleotide sequences from *S. aureus* clinical and reference strains were translated using the *in silico* web page ^3^; the amino acid sequences of the SapS protein were used for analyzing the N-terminal signal peptide domain, its cleavage sequence, and its type of protein secretion system category using the SignalP 5.0 server platform ^12^. The subcellular location of SapS for Gram-positive bacteria was analyzed on the Gpos-mPloc platform ^13^. The bipartite signature of aspartate (DDDD) motifs of class C non-specific acid phosphatases was searched using BLAST tools ^14^.

### Isolation and concentration of protein extracts from bacterial culture media

All bacterial strains were grown in a casein peptone-soymeal peptone broth (CASO, Merck) for 18 h at 37 °C under constant shaking. Bacteria were then separated by centrifugation at 10,000*g* for 10 minutes at 4 °C; supernatants were sterilized by filtration through 0.22 pm membranes (S-Pak Millipore), aliquoted, and stored at -80 °C. Supernatant (or uninoculated CASO culture medium) protein extracts were obtained by mixing one volume of each supernatant with four volumes of cold (-20 °C) ultrapure acetone (Merck EM Science); the protein was allowed to precipitate at -75 °C for one hour, recovered by centrifugation at 14,000*g* for 15 minutes at 4 °C, dried at 4 °C, and resuspended in 1.0 ml of 0.1 M sodium acetate solution, pH 5.0 (buffer A). Aliquots (200 pl) were stored at -80 °C. Protein was measured using detergent compatible colorimetric technique (DC Protein Assay, BioRad).

### Acid phosphatase activity

Acid phosphatase activity in bacterial culture media was measured as described by Golovan *et al.*^15^ with *p*-nitro-phenyl phosphate (*p*-NPP disodium salt, hexahydrate, Sigma Chem). Reaction mixtures (100 pl) containing 10 or 20 pl of the protein extracts and 200 pl of 10 mM p-NPP in buffer A were incubated for 30 minutes at 37 °C; reactions were stopped with 100 pl of 1.0 M NaOH solution, and the *p*-nitrophenol (p-NP) formed was measured by absorbance at 405 nm (Beckman DU 800 spectrophotometer). Specific acid phosphatase activity was defined as pmoles *p*-NP/min/mg protein. All measurements were made in triplicate.

To 10 pl of protein extracts, we added one of the following phosphatase inhibitors: 20 mM dibasic dihydrate sodium tartrate, 10 mM sodium fluoride, 2 mM ammonium molybdate tetrahydrate, and 10 mM sodium ortho-vanadate dissolved in buffer A. Reaction mixtures were adjusted to a final volume of 100 pl and incubated initially at 37 °C for 15 minutes. Then, 200 pl of 10 mM *p*-NPP were added and reaction mixtures were further incubated for 30 minutes at 37 °C. The reaction was stopped with 100 pl of 1.0 M NaOH solution. Specific acid phosphatase activity was calculated as mentioned above.

### Identification of extracellular SapS in protein extracts of clinical and reference strains culture media supernatants

SapS presence in protein extracts from culture media supernatants was analyzed by zymography. We seeded 10 pl per well of each clinical and reference protein extract, the noninoculated culture medium together and a standard pre-stained protein molecular weight marker (Precision Plus Protein, All Blue Prestained Protein Standards, BioRad). Protein extracts were run in a 12% sodium dodecyl sulfate polyacrylamide gel electrophoresis (SDSPAGE) under nonreducing conditions, basically as described by Hamilton *et al.*^16^.

After electrophoresis, SDS was removed by washing gels twice with deionized water and once with 1.0% Triton X-100 solution. Then, gels were incubated in renaturing buffer (100 mM Tris HCl, 2 mM MgSO4^6H_2_O, 1% Triton X-100 pH 7.0) for 15 minutes at room temperature and equilibrated in buffer A. Acid phosphatase activity was assessed by adding 0.1 % of a-naphthyl phosphate and 0.2% of Fast Garnet GBC (Sigma. Chem) to the buffer and further incubating at 37 °C for 45 minutes. a-naphthol appeared as a brownish-red precipitate on the protein band with phosphatase activity. The reaction was stopped by washing the gel twice with deionized water. Protein bands on parallel gels, run under similar conditions, were stained with Coomassie Brilliant Blue R-250 (BioRad).

### SapS specificity for phospho-L-amino-acids and the effect of phosphatase inhibitors

SapS substrate specificity for O-phospho-aminoacids was also determined by zymography according to Slotnick and Gottlieb ^17^, with modifications. After SDS-PAGE and renaturation as described above, gel were incubated at 37°C for 45 minutes in 10 ml of Buffer A solution with 4 mM of each of the following substrates: O-phospho-L-serine, O-phospho-L-threonine, and OphosphoLtyrosine (Sigma Chem). Then, gels were washed twice with deionized water. The released inorganic phosphate was detected by gel incubation with a mixture of 0.045% malachite green (oxalate salt, Sigma Chem) and 4.2% ammonium molybdate for 10 minutes at room temperature. The reaction was stopped with two washes of deionized water followed by 5% of acetic acid solution. A positive phosphatase reaction was a green precipitate on the SapS protein band stained with Coomassie Brilliant Blue in a parallel gel, run under similar conditions.

Similarly, phosphatase inhibitors for SapS catalysis were tested by zymography. Renatured gels were incubated at 37 °C for 30 minutes in 10 ml of a 10 mM sodium tartrate, sodium fluoride, sodium ortho-vanadate, or ammonium molybdate (Sigma Chem) solution. Gels were washed once in buffer A and further incubated at 37 °C for another 30 minutes in 10 ml of 0.1% a-naphthyl phosphate or 4 mM of each of the three O-phospho-amino acids as substrates. The corresponding reaction products, a-naphthol and released phosphate, were stained as described above with 0.2% Fast Garnet GBC or malachite green/ammonium molybdate, respectively.

### Statistical analysis

The statistical significance of differences among the means of acid phosphatase measurements with and without enzyme inhibitors was determined with an ANOVA test and a comparative Tukey post-hoc test using the GraphPad Prism 5.0 software. Significance was set at p<0.05.

## Results

### *SapS* gene is present in the genomes of isolated *S. aureus* clinical strains from chronically osteomyelitic patients

Using the primers designed for *S. aureus* 154 ^8^, we detected the presence of the complete *SapS* gene as a single 891 bp-long fragment in the 12 clinical strains and *S. aureus* ATCC-6538, but not in *S. epidermidis* ATCC-12228. With SapS2 primer set we obtained a single 805 bp-long fragment that matched the nucleotide sequence of the extracellular mature peptide with acid phosphatase activity. These results show that the *SapS* gene and the nucleotide sequence encoding the extracellular phosphatase are present in the clinical and the reference *S. aureus* strains, but not in *S. epidermidis* ATCC-12228.

### SapS gene is present in S. aureus strains but not in the coagulasenegative Staphylococcus strains from the database with complete bacterial genomes.

**Figure 1:**
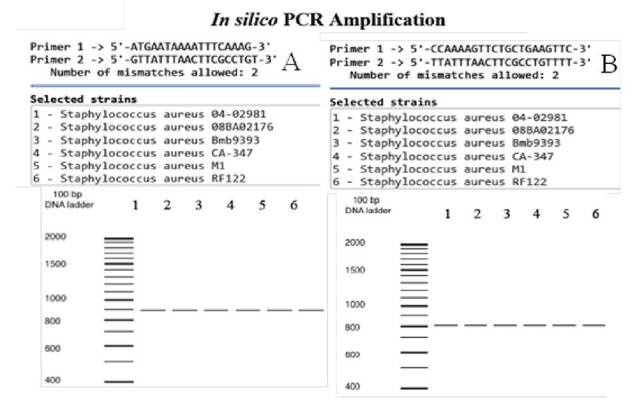
*In silico* PCR amplifications. A. *SapS* gene amplifications using the oligonucleotide primers described by du Plessis, *et al*. ^8^. B. Amplifications of the *SapS* gene region encoding for the mature extracellular acid phosphatase (SapS2) using our set of oligonucleotide primers. In both cases, DNA templates were from the 49 *S. aureus* strains and the 11 coagulase-negative *Staphylococcus* strains, and from the plasmids housed in the database with the *in silico* analysis of bacterial genomes ^10^. Amplifications of six coagulase-positive *S. aureus* strains are shown as examples. No amplifications were found for coagulase-negative strains.

*In silico* PCR confirmed the presence of the *SapS* gene only in the *S. aureus* species. Using the original primer set ^8^, the 892 bp-long fragment was obtained for all the 49 *S. aureus* strains tested with the *in silico* PCR (figure 1A) and none for any of the 11 coagulase-negative Staphylococcus strains. Similarly, with the SapS2 primers, only a single 800 bp-fragment was obtained for *S. aureus* strains (figure 1B); no amplifications were obtained with either primers for any of the plasmids contained in the complete bacterial genomes database.

### *The SapS gene nucleotide and amino acid sequences reveal the presence of the protein* SapS *catalytic domain and the signal peptide cleavage site.*

The multiple nucleotide sequence analysis performed with the *SapS* sequences of *S. aureus* 154 ^8^, the 12 clinical *S. aureus* strains, and the *S. aureus* ATCC-6538 reference strain showed an identity percentage of 92-96% (table 1) suggesting that *SapS* gene from the assessed *S. aureus* strains are homologous. The built phylogenetic tree (data not shown) revealed the closeness among the sequences implying they could share a common ancestor.

**Table 1:** Identity percentage of nucleotide sequence similarities between the SapS gene secuences of our 12 clinical *Staphylococcus aureus* isolates, the American Type Culture Collection *Staphylococcus aureu*s strain, code ATCC-6538; and the *Staphylococcus aureus* 154 strain (accession number AY61973.1) ^5^.

Clinical S. aureus isolates	105 IMO	76 IQM	17 CPJ	AY061973.1*	ATCC 6538*	54 SL	107 FMR	101 AOC	69 GGT	88 VTM	68 FFC	92 HM	93 EMC	89 RTC
105 IMO	100.0	95.01	94.73	96.28	94.48	95.30	95.19	94.20	94.77	92.48	92.37	91.93	91.98	91.68
76 IQM	95.01	100.0	96.76	95.89	96.29	96.76	95.95	96.42	96.30	92.06	93.34	92.60	93.67	93.67
17 CPJ	94.73	94.76	100.0	99.30	98.17	96.34	97.26	96.69	96.80	92.30	93.64	92.24	93.40	93.29
AY61973.1*	96.28	95.89	99.30	100.0	99.07	97.68	98.38	98.03	97.92	92.78	94.97	93.65	95.19	94.84
ATCC6538*	94.48	96.29	98.17	99.07	100.0	96.10	97.02	96.56	96.45	93.41	93.39	92.09	93.26	93.15
54 SL	95.30	96.76	96.34	97.68	96.10	100.0	96.46	96.23	96.80	92.49	93.63	92.81	93.97	94.09
107 FMR	95.19	95.95	97.26	98.38	97.02	96.46	100.0	96.70	97.27	93.08	93.53	92.70	93.99	93.99
101 AOC	94.20	96.42	96.69	98.03	96.56	96.23	96.70	100.0	96.60	92.17	92.55	92.48	96.55	93.00
69 GGT	94.77	96.30	96.80	97.92	96.45	96.80	97.27	96.60	100.0	92.62	93.23	92.94	93.56	93.68
88 VTM	92.48	92.06	92.00	92.78	93.41	92.49	93.08	92.17	92.62	100.0	93.16	93.36	94.19	94.19
68 FFC	92.37	93.34	93.64	94.97	93.39	93.63	93.53	92.55	93.23	93.16	100.0	94.72	93.39	92.62
92 HM	91.93	92.60	92.24	93.65	92.09	92.81	92.70	92.48	92.94	93.36	94.72	100.0	95.27	95.29
93 EMC	91.98	93.67	93.40	95.19	93.26	93.97	93.99	92.65	93.56	94.19	93.39	95.27	100.0	92.72
89 RTC	91.68	93.67	93.29	94.84	95.15	94.09	93.99	93.00	93.68	94.19	92.62	95.29	97.72	100.0

*Known sequences

The amino acid sequence analysis of the *SapS* gene on the SignalP 5.0 server showed the presence of the signal peptide in the N-terminal amino acid sequence (27 - STAFAKSSAEVQQ - 39) in the 12 clinical *S. aureus* strains (table 2) as previously reported ^7^. It included the recognition site for signal peptide cleavage between residues alanine and lysine in the positions 31 and 32 (AK) respectively**.**

**Table 2:** Conserved and relevant SapS amino acid sequences in Staphylococcus. aureus clinical strains.

***S. aureus* strain**	Signal peptide	Signature motif A	Signature motif B
154 (5)	27-STAF**AK**SSAEVQQ-39	101-ALDLDETVLDNSPY-114	213-LVMLFGDNLLDF-224
17 CPJ	27-STAF**AK**SSAEVQQ-39	101-ALDLDETVLDNSPY-114	213-LVMLFGDNLLDF-224
54 SL	27-STAF**AK**SSAEVQQ-39	101-ALDLDETVLDNSPY-114	213-LVMLFGDNLLDF-224
68FFC	27-STAF**AK**SSAEVQQ-39	101-ALDLDETVLDNSPY-114	213-LVMLFGDNLLDF-224
69 GGT	27-STAF**AK**SSAEVQQ-39	101-ALDLDETVLDNSPY-114	213-LVMLFGDNLLDF-224
76 IQM	27-STAF**AK**SSCSCST-39	101-ALDLDETVLDNSPY-114	213-LVMLFGDNLLDF-224
88 VTM	27-STAF**AK**SSGEVQQ-39	101-ALDLDETVLDNSPY-114	213-LVMLFGDNLLDF-224
89 RTC	27-STAF**AK**SSAEVQQ-39	101-ALDLDETVLDNSPY-114	213-LVMLFGDNLLDF-224
92 HM	27-STAF**AK**SSAEVQQ-39	101-ALDLDETVLDNSPY-114	213-LVMLFGDNLLDF-224
93 EMC	27-STAF**AK**SSAEVQQ-39	101-ALDLDETVLDNSPY-114	213-LVMLFGDNLLDF-224
101 AOC	27-STAF**AK**SSAEVQQ-39	101-ALDLDETVLDNSPY-114	213-LVMLFGDNLLDF-224
105 IMO	27-STAF**AK**SSAEVQQ-39	101-ALDLDETVLDNSPY-114	213-LVMLFGDNLLDF-224
107 FMR	27-STAF**AK**SSAEVQQ-39	101-ALDLDETVLDNSPY-114	213-LVMLFGDNLLDF-224
ATCC 6538	27-STAF**AK**SSAEVQQ-39	101-ALDLDETVLDNSPY-114	213-LVMLFGDNLLDF-224

The predictive analysis on the Gpos-mLoc server suggested that SapS is a typical extracellular protein belonging to the *S. aureus* secretome ^18^. Its bipartite motifs A (101 - ALDLDETVLDNSPY - 114) and B (213 - LVMLFGDNLLDF - 224), and the associated four invariant residues of aspartate ^19^ - with dephosphorylating enzymatic activity of class “C” NSAPs ^20^ - were also found to be highly conserved in our 12 clinical *S. aureus* strains (table 2).

### SapS resistance to sodium tartrate and sodium fluoride, and sensitivity to sodium ortho-vanadate and ammonium molybdate

**Figure 2:**
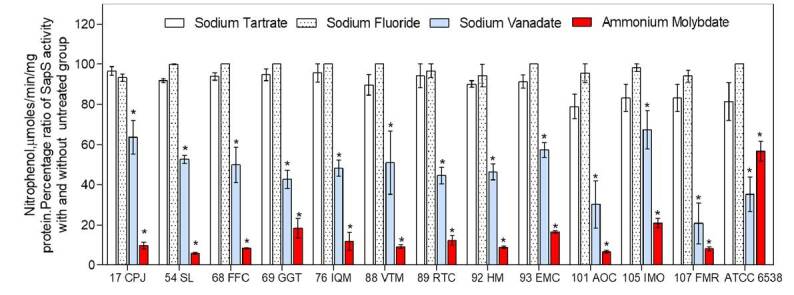
Percentageof *appS* activityinthepresenceandabsenceof phosphatasesinhibitors. *SppS* activitywasassessed from protein extracts of culture media of clinical strains; 10 mM of*p*-nitro-phenyl-phosphate was used as substrate. Assays were run in triplicate from three independent experiments; results are expressed as mean ± standard error of the mean. Statistical analysis was performed using ANOVA and significance was set at *p<0.05.

**Figure 3:**
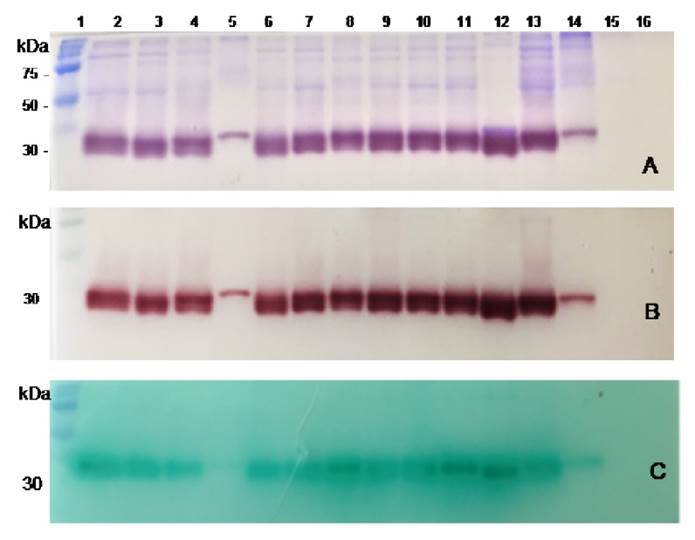
Non-reducing gel electrophoresis and zymographic analysis of protein extracts obtained from the culture media of clinical bacterial strains, reference strains, and the sterile culture medium (SCM). A. Coomassie Brilliant Blue for protein staining. B. Brown red a-naphthol-Fast Garnet GBC complex for SapS activity assessment, and C. Phosphate/green malachite/ ammonium molybdate complex for SapS phosphatase activity on O-phospho- L-tyrosine. Lanes from left to right: 1, pre-stained protein molecular weight; 2 13, clinical bacterial strains; 17, CPJ, 54 SL, 68 FFC, 69 GGT, 76 IQM, 88 VTM, 89-RTC, 92-HM, 93-EMC,101-AOC,105-IMO,107-FMR; 14, *Stpphylococcus pureus* ATCC-6538 strain; 15, *Stpphylococcus epidermidis* ATCC-12228; 16, SCM.

The average acid phosphatase-specific activity was 25.35 mmoles of *p*-nitrophenol per minute and per mg of protein at pH 5.0 (figure 2) in the extracts from the culture media of the 12 *S. aureus* clinical strains incubated with *p*-nitro-phenyl phosphate as substrate. *Staphylococcus aureus* ATCC- 6538 showed the lowest level of activity (4.5 mmoles *p*-NP/min/mg of protein) and *S. epidermidis* ATCC-12228 strain showed no activity at all. Moreover, this extracellular acid phosphatase activity was resistant to inhibition by both sodium tartrate, a general phosphatase inhibitor, and sodium fluoride, an inhibitor of acid phosphatases (figure 3). However, this activity was inhibited by sodium ortho-vanadate (~ 50%) and ammonium molybdate (80-95%) (figure 2).

### SapS is present in semi-purified culture media supernatant protein extracts of S. aureus clinical strains.

Non-reducing SDS-PAGE of partially purified culture media supernatant protein extracts of *S. pureus* clinical strains and *S. pureus* ATTC-6538 (figure 3A) showed remarkably similar extracellular protein profiles (Coomassie brilliant blue-staining) ranging from 30 to 100 kDa. Zymograms analysis showed only one extracellular protein band with phosphatase activity for clinical *S. pureus* and *S. pureus* ATCC-6538 strains, as revealed by the appearance of a-naphthol at the same migration distance as the protein band run in a parallel gel, stained with Coomassie brilliant blue. Both bands showed an approximate molecular mass of 30 kDa (figures 3A and 3B) strongly suggesting their identity with SapS. The protein extract from the *S. epidermidis* ATCC-12228 (figures 3A and 3B, lane 15) showed no 30 kDa- protein band, nor associated enzymatic activity.

### SapS exhibits O-phospho-L-tyrosine phosphatase activity and is sensitive to sodium vanadate and ammonium molybdate.

The zymograms analysis of the culture media protein extracts of all *S. aureus* clinical strains and *S. aureus* ATTC-6538 strain in the presence of Ophospho-amino-acids showed a green malachite/molybdate/phosphate complex precipitate at the same migration distance as the 30 kDa observed in the Coomassie stained-gel and the brownish-red a-naphthol/Fast Garnet GBC precipitate staining. These results confirmed SapS phosphatase activity (figure 3C).

However, SapS showed O-phospho-L-tyrosine phosphatase activity while neither OphosphoLserine, nor O-phospho-L-threonine was dephosphorylated. It appears, therefore, that SapS and the o-phospho-L-tyrosine phosphatase are the same enzyme.

Furthermore, in another set of parallel zymograms including one of the four phosphatase inhibitors mentioned, we found that the green malachite/ molybdate/phosphate complex precipitate, formed after O-phospho-tyrosine dephosphorylation, was resistant to sodium tartrate and sodium fluoride, but did not appear in the presence of sodium vanadate and ammonium molybdate.

## Discussion

Bone destruction in chronic staphylococcal osteomyelitis is due to an inflammatory process induced by the expression of many *S. aureus* exoproteins whose number and function remain unresolved ^1^. Notably, since the initial characterization of SapS in the *S. aureus* 154 strain, the presence of the *SapS* gene and the activity of the coding extracellular acid phosphatase have not been examined in the context of bone infections caused by *S. aureus*. Here we have identified and sequenced *SapS* gene in our 12 clinical strains and 49 others from a database containing *in-silico* analysis of complete bacterial genomes. We also found and characterized SapS in the partially purified protein extracts from the culture media of the same clinical strains.

The *SapS* gene and the coding region for the extracellular mature peptide ^8^ found in our clinical strains and in the database’s genomes (*S. aureus* strains clinically relevant of diverse origins) suggest that this gene could be part of an *S. aureus* “core” genome shared by all the strains and likely encodes for functions related to the bacteria basic cellular nutrition ^20^. *S. aureus* “core” genome has been studied in large groups of *S. aureus* strains and no significant genotypic differences have been found between strains from asymptomatic carriers and patients with invasive infections ^21^.

NSAP, the superfamily of acid phosphatases presents in prokaryotes and eukaryotes, exhibit non-specific activity on many structurally unrelated phosphoesters. Prokaryote NSAPs are usually grouped into three classes, namely A, B, and C, based on their amino acid sequence relatedness and the highly conserved bipartite sequences of their signature motif of aspartate (DDDD); the two couples of invariant aspartate residues present in each domain are essential for its enzymatic activity ^7^.

Our *SapS* gene amino acid sequence analysis corroborated the presence of this bipartite domain in the 12 chronic osteomyelitis-causing *S. aureus* strains and others of clinical importance. We further disclosed one of the four known types of staphylococcal Nterminal signal peptide domains for SapS. The previous result suggest SapS is exported, since this type of N-terminal signal peptide domain directs proteins to a particular transport pathway ^22^. Moreover, its predictive analysis indicated, with high probability (95.56%), that SapS could be released as an extracellular class C non-specific acid phosphatase lipoprotein with a protein component of roughly 30 kDa. In that case, SapS would be part of the *S. aureus* secretome ^23^.

The N-terminal signal peptide domain feature differs from the targeted subcellular location anticipated for other NSAP class C of pathogenic bacteria like *Clostridium perfringens* that are devoid of an N-terminal consensus lipoprotein-signal peptide-like motif ^24^; or for *Flavobacterium meningosepticum, Bacillus anthracis*, *Helicobacter pylori*, *Streptococcus pyogenes*, and *Haemophilus influenza,* whose acid phosphatase N-terminal signal peptide sequences show a 98.75% probability of being membranebound lipoprotein-type enzymes and only a 1.22% probability of being extracellular phosphatases as expected for SapS.

The biochemical analysis of SapS activity, in partially purified protein extracts from the culture media of the clinical strains of *S. aureus,* showed the presence of the 30 kDa protein with a highly selective phosphatase activity for O-phospho-L-tyrosine, and sensitive to vanadate and molybdate. Notably, these characteristics are shared with the extracellular 28 kDa type of NSAP class C: SapM, and the low molecular weight-phosphorylated-tyrosine protein phosphatases (LMW-PTP) MptpA of pathogenic *Mycobacterium tuberculosis*, which are proven virulence factors ^23^^,^^25^.

*Staphylococcus aureus* genome contains all the information required for its development and functioning, including an arsenal of virulence genes coding for proteins involved in the adherence and colonization of infected tissues along with their immune evasive properties ^26^. Some of these proteins are well- known virulence factors with newly identified mechanisms of action, while the functional characterization of newer ones is currently under investigation ^27^.

Considering the structural and functional similarities of the SapS from our *S. aureus* osteomyelitis-causing strains with the NSAPs class C and LMW-PTPs, which are well-known virulence factors in various pathogenic strains, we suggest that SapS may also be a virulence factor in *S. aureus*. Since inorganic phosphate is a vital element for *S. aureus* metabolism, it is not freely available, and is limited during infection, SapS might be the extracellular processing enzyme required by the bacteria to extract inorganic phosphates from host organophosphates (phosphorylated nucleotides, sugars, and amino acids). These inorganic phosphates are then imported through the inorganic phosphate transporters to ensure bacteria survival and probably helps to enhance bone damage during infection ^28^^,^^29^.

Proving that SapS is in fact a virulence factor will require a detailed series of studies aimed at establishing the precise physiological function of the purified enzyme in the metabolism of bone tissue and cells. These are needed to determine if this newly identified and partially characterized NASP class C contributes to the pathogenicity of *S. aureus* in chronic osteomyelitis.
